# NFAT isoforms play distinct roles in TNFα-induced retinal leukostasis

**DOI:** 10.1038/srep14963

**Published:** 2015-11-03

**Authors:** Colin A. Bretz, Sara R. Savage, Megan E. Capozzi, Sandra Suarez, John S. Penn

**Affiliations:** 1 Vanderbilt University Medical Center, Department of Cell and Developmental Biology, Nashville, TN, USA; 2 Vanderbilt University Medical Center, Department of Pharmacology, Nashville, TN, USA; 3 Vanderbilt University Medical Center, Department of Molecular Physiology and Biophysics, Nashville, TN, USA; 4 Vanderbilt University Medical Center, Department of Ophthalmology and Visual Sciences, Nashville, TN, USA

## Abstract

The objective of this study was to determine the role of individual NFAT isoforms in TNFα-induced retinal leukostasis. To this end, human retinal microvascular endothelial cells (HRMEC) transfected with siRNA targeting individual NFAT isoforms were treated with TNFα, and qRT-PCR was used to examine the contribution of each isoform to the TNFα-induced upregulation of leukocyte adhesion proteins. This showed that NFATc1 siRNA increased *ICAM1* expression, NFATc2 siRNA reduced *CX3CL1*, *VCAM1*, *SELE*, and *ICAM1* expression, NFATc3 siRNA increased *CX3CL1* and *SELE* expression, and NFATc4 siRNA reduced *SELE* expression. Transfected HRMEC monolayers were also treated with TNFα and assayed using a parallel plate flow chamber, and both NFATc2 and NFATc4 knockdown reduced TNFα-induced cell adhesion. The effect of isoform-specific knockdown on TNFα-induced cytokine production was also measured using protein ELISAs and conditioned cell culture medium, and showed that NFATc4 siRNA reduced CXCL10, CXCL11, and MCP-1 protein levels. Lastly, the CN/NFAT-signaling inhibitor INCA-6 was shown to reduce TNFα-induced retinal leukostasis *in vivo*. Together, these studies show a clear role for NFAT-signaling in TNFα-induced retinal leukostasis, and identify NFATc2 and NFATc4 as potentially valuable therapeutic targets for treating retinopathies in which TNFα plays a pathogenic role.

Diabetic retinopathy (DR) is one of the leading causes of blindness in working age adults, affecting nearly 35% of diabetic patients worldwide^1^,^2^. Inflammation is a major component of DR, and a number of inflammatory cytokines known to promote diabetes-induced retinal pathology are elevated in the vitreous of patients with DR^3^,^4^,^5^,^6^. One such cytokine is tumor necrosis factor-alpha (TNFα), which has been established as a critical driver of retinal leukocyte adhesion in diabetic animals^7^,^8^,^9^. Increased numbers of adherent leukocytes are also found in the retinal vasculature of diabetic patients, and the adhesion of these myeloid-derived cells to the vascular endothelium is commonly referred to as leukostasis^10^,^11^. A common pathogenic feature of DR, leukostasis can contribute to pathology and progression through a variety of mechanisms. Adherent leukocytes can secrete proteolytic enzymes and inflammatory cytokines that contribute to blood-retinal barrier breakdown, increased vascular permeability, and retinal edema, and can also occlude retinal capillaries leading to focal ischemia and an increase in the vasoactive factors that drive pathologic neovascularization^12^,^13^,^14^.

One way in which TNFα is believed to contribute to retinal leukostasis is by activating endothelial cells and inducing their expression of leukocyte adhesion proteins chemokine (C-X3-C motif) ligand 1 (CX3CL1), vascular cell adhesion molecule 1 (VCAM1), E-selectin, and intercellular adhesion molecule 1 (ICAM1), which are involved in the capture, tethering, and firm adhesion of leukocytes to the vascular endothelium^15^,^16^,^17^,^18^,^19^,^20^,^21^,^22^. TNFα also stimulates endothelial cell production of cytokines such as chemokine (C-X-C motif) ligand 10 (CXCL10), chemokine (C-X-C motif) ligand 11 (CXCL11), monocyte chemoattractant protein-1 (MCP-1), and interleukin-6 (IL-6), which have established roles in leukocyte recruitment^8^,^23^,^24^,^25^,^26^. We have recently demonstrated that TNFα is a strong inducer of these gene products in human retinal microvascular endothelial cells (HRMEC), and that nuclear factor of activated T-cell (NFAT) family transcription factors regulate a subset of these genes related to leukocyte cell adhesion and recruitment^23^. NFAT is a family of five transcription factors, four of which (NFATc1-c4) are regulated by binding to the phosphatase calcineurin (CN) at a conserved regulatory domain^27^,^28^. The fifth isoform, NFAT5, does not contain a CN regulatory domain and is not involved in the CN/NFAT signaling pathway. The small molecule Inhibitor of NFAT-Calcineurin Association-6 (INCA-6) effectively blocks CN activation of the four CN-dependent isoforms, and this has been shown to reduce TNFα-induced expression of *CX3CL1*, *VCAM1*, *CXCL10*, and *CXCL11* in HRMEC^29^,^30^.

Taken together, this evidence suggests that NFAT-signaling may play an important role in TNFα-induced retinal leukostasis. However, the precise roles played by individual NFAT isoforms in these contexts and whether NFAT inhibition has a functional effect on TNFα-induced leukocyte adhesion remains unknown. To address this, the present study evaluates the effects of *in vitro* NFAT isoform-specific siRNA knockdown on TNFα-induced leukocyte adhesion protein expression, peripheral blood mononuclear cell adhesion, and cytokine secretion, as well as the *in vivo* effect of INCA-6 mediated CN/NFAT-signaling inhibition on TNFα-induced retinal leukostasis.

## Results

### Effect of NFAT isoform-specific knockdown on TNFα-induced adhesion protein expression

TNFα-stimulation (1 ng/ml for 4 hrs) of HRMEC resulted in increased expression of the leukocyte adhesion proteins CX3CL1, VCAM1, E-Selectin, and ICAM1 (Table 1). All of these have been identified as NFAT-signaling targets in an endothelial cell context^23^,^31^,^32^,^33^. In order to determine what role(s) individual NFAT isoforms play in this expression, HRMEC were transfected with either control or isoform-specific siRNA, and the effects on TNFα-induced expression were evaluated using qRT-PCR.

**Figure 1:**
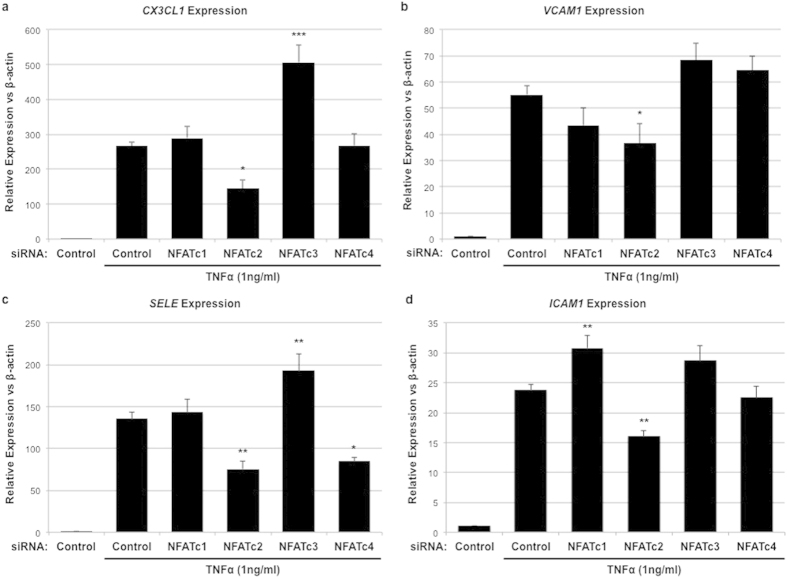
The effect of NFAT isoform-specific siRNA knockdown on TNFα-induced expression of leukocyte adhesion proteins. HRMEC transfected with either control or NFAT isoform-specific siRNA were treated with TNFα and total RNA was then collected for qRT-PCR analysis. Each bar represents the mean ± SEM (n = 9). *p < 0.05, **p < 0.01, ***p < 0.0001.

NFATc2 siRNA knockdown reduced TNFα-induced expression of *CX3CL1* by 45.6% (p = 0.0238, Fig. 1a), while NFATc3 knockdown increased the expression by 90.3% (p < 0.0001). NFATc1 and NFATc4 knockdown did not affect TNFα-induced expression of *CX3CL1*. TNFα-induced expression of *VCAM1* was inhibited 34.9% by NFATc2 knockdown (p = 0.0468, Fig. 1b), while knockdown of NFATc1, NFATc3, and NFATc4 had no effect on TNFα-induced *VCAM1* expression. NFATc2 and NFATc4 knockdown reduced TNFα-induced *SELE*, which encodes the E-selectin protein, expression by 44.3% (p = 0.004 Fig. 1c) and 37.3% (p = 0.0121) respectively, while NFATc3 knockdown increased expression by 42.5% (p = 0.0033). NFATc1 knockdown did not effect TNFα-induced expression of *SELE*. NFATc1 knockdown did increase TNFα-induced *ICAM1* expression by 29.3% (p = 0.0091 Fig. 1d), while NFATc2 knockdown reduced it by 32.6% (p = 0.0077). Knockdown of NFATc3 and NFATc4 had no effect on TNFα-induced *ICAM1* expression.

### Effect of NFAT isoform-specific knockdown on TNFα-induced PBMC adhesion

**Figure 2:**
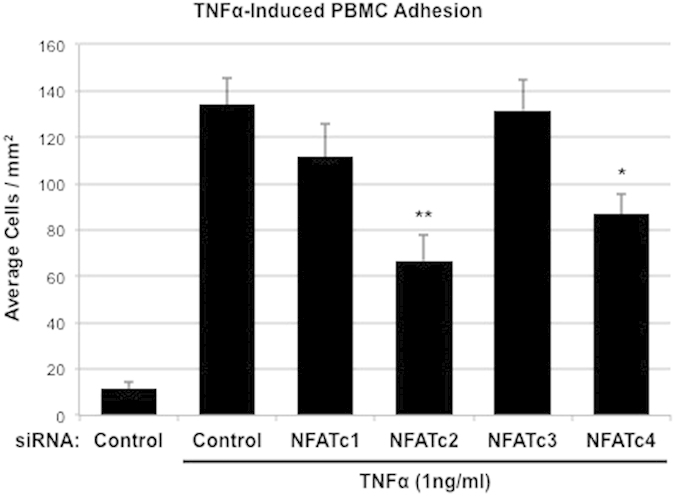
The effect of NFAT isoform-specific siRNA knockdown on TNFα-induced PBMC adhesion. HRMEC monolayers transfected with either control or isoform-specific siRNA were treated with TNFα and then grown in a confluent monolayer on the floor of a parallel plate flow chamber. PBMCs were flowed over the monolayer for 7 min. Non-adherent cells were washed off and the remaining cells were counted. Each bar represents the mean ± SEM (n = 5). *p < 0.05, **p < 0.01.

In order to assess the functional effects of isoform-specific changes in TNFα-induced leukocyte adhesion protein expression, HRMEC monolayers were again transfected with either control or isoform-specific siRNA and evaluated using a parallel plate flow chamber (PPFC) assay. TNFα treatment (1 ng/ml for 4 hrs) of monolayers transfected with control siRNA induces an increase in the number of PBMC that adhere to the endothelial monolayers compared to non-stimulated controls (Table 1). Monolayers transfected with NFATc2 and NFATc4 siRNA reduced TNFα-induced PBMC adhesion by 55% (p = 0.0013, Fig. 2) and 38.4% (p = 0.0289), respectively, while transfection with NFATc1 and NFATc3 siRNA had no significant effect on TNFα-induced cell adhesion.

### Effect of NFAT isoform-specific knockdown on TNFα-induced cytokine production

TNFα-stimulation of HRMEC also results in increased expression of several soluble inflammatory cytokines, including *CXCL10*, *CXCL11*, *MCP-1*, and *IL-6*^23^. These have all been identified as NFAT signaling targets in endothelial cells^32^,^34^. In order to determine what role individual NFAT isoforms play in the production of these soluble cytokines, transfected HRMEC were treated with TNFα (1 ng/ml for 6 hrs) and conditioned media were collected and assayed using ELISA to determine TNFα-induced cytokine production (Table 1).

**Figure 3:**
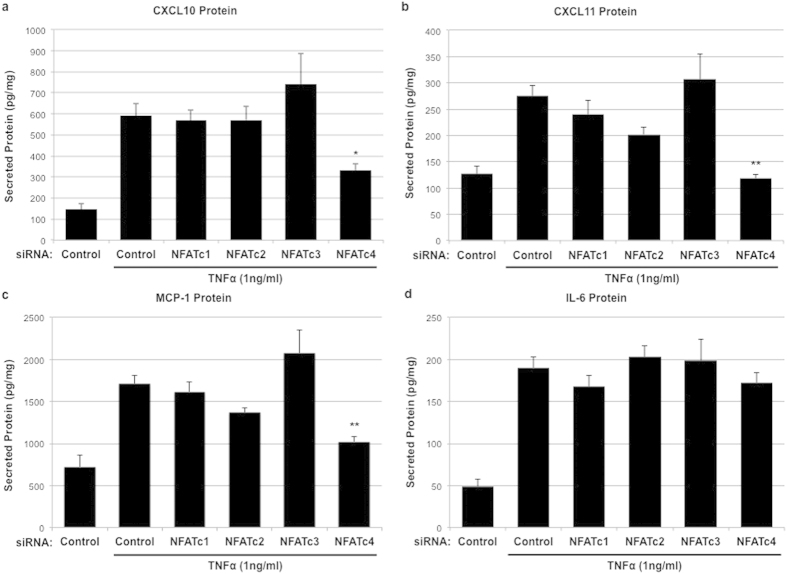
The effect of NFAT isoform-specific siRNA knockdown on TNFα-induced cytokine production. HRMEC transfected with either control or NFAT isoform-specific siRNA were treated with TNFα and conditioned media was then collected and analyzed for secreted cytokines using ELISA. Each bar represents the mean ± SEM (n = 9). *p < 0.05, **p < 0.01.

Transfection with NFATc4-specific siRNA was the only treatment that affected TNFα-induced cytokine production, reducing CXCL10, CXCL11, and MCP-1 protein in conditioned media by 58.1% (p = 0.0369, Fig. 3a), 105.7% (p = 0.0004, Fig. 3b), and 69.67% (p = 0.0066, Fig. 3c) respectively. Knockdown of NFATc1, NFATc2, and NFATc3 had no effect on TNFα-induced CXCL10, CXCL11, and MCP-1 protein levels in media, and the induction of IL-6 was unaffected by transfection with any of the NFAT isoform-specific siRNA (Fig. 3d).

### Effect of NFAT-inhibition on TNFα-induced retinal leukostasis

**Figure 4:**
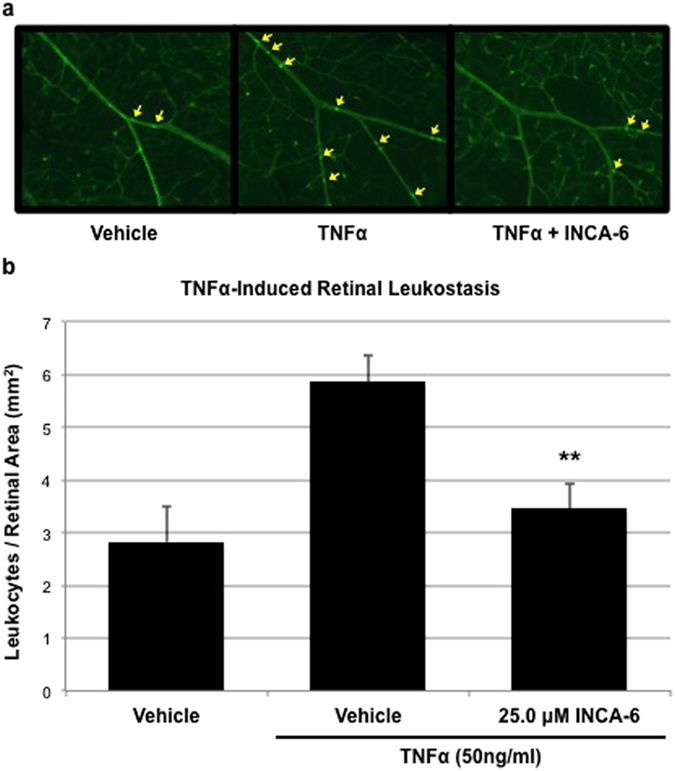
INCA-6 inhibits TNFα-induced retinal leukocyte adhesion. C57BL/6 mice received intravitreal injections of vehicle, TNFα, or TNFα plus INCA-6, followed by perfusion with saline to flush non-adherent cells and infusion with concanavalin A to stain adherent cells. Adherent leukocytes were then counted in flat-mounted retinas. Representative images of adherent leukocytes following treatment are provided and quantification of adherent leukocytes is reported as per mm^2^. Each bar represents the mean ± SEM (for Vehicle, n = 5; for TNFα + Vehicle, n = 9; for TNFα + INCA-6, n = 6). **p < 0.01.

Lastly, as a proof of concept that NFAT-inhibition may be a viable chemotherapeutic strategy to address TNFα-induced retinal leukostasis, the effect of non-isoform-specific CN/NFAT inhibition was evaluated *in vivo* using the broad-spectrum NFAT inhibitor INCA-6. Intravitreal injection of TNFα (50 ng/ml) significantly induced retinal leukocyte adhesion compared to control after 6 hrs (p = 0.0023, Fig. 4), and co-injection of INCA-6 (25 μM) reduced this effect by 79.2% (p = 0.0089).

## Discussion

This study is the first to examine the role of individual NFAT isoforms in the context of retinal leukostasis. We have previously examined the genome-wide transcriptional effects of TNFα-stimulation on HRMEC, and used INCA-6 treatment to identify a general role for CN/NFAT-signaling in TNFα-induced expression of a subset of genes related to leukocyte adhesion and chemoattraction^23^. INCA-6 competitively binds to the discrete NFAT-binding site of CN, and serves as a general inhibitor of NFATc1, NFATc2, NFATc3, and NFATc4, without altering CN phosphatase activity^29^,^30^. Therefore, while NFAT-signaling was previously shown to play a role in the endothelial cell response to TNFα-stimulation, the role of individual NFAT isoforms remained unknown.

To address this, we transfected HRMEC with isoform-specific siRNA and evaluated the effect of individual NFAT-isoform knockdown on TNFα-induced gene expression. The first two targets analyzed were *CX3CL1* and *VCAM1*, which were both shown to be inhibited by INCA-6 treatment in our previous study^23^. CX3CL1 is an inflammatory cytokine that, as a soluble factor, aids in leukocyte recruitment to areas of inflammation, and in its more common membrane-bound form is important for leukocyte tethering and adhesion^15^,^16^,^17^. VCAM1 is a well-characterized cell adhesion molecule involved in the firm adhesion of leukocytes to vascular endothelium^18^,^20^,^21^,^22^. Isoform-specific siRNA revealed that NFATc2 knockdown negatively regulated both *CX3CL1* and *VCAM1* expression, and that NFATc3 knockdown increased TNFα-induced upregulation of *CX3CL1*. The latter finding shows that individual NFAT isoforms can play counteractive roles in endothelial cell activities, and highlights the importance of evaluating isoforms individually.

Given these initial findings, we expanded our evaluation to look at the role of NFAT isoforms in TNFα-induced *SELE* and *ICAM1* expression. *SELE* is the gene that codes for E-selectin, and both E-selectin and ICAM1 are adhesion proteins known to mediate TNFα-induced leukocyte adhesion^18^,^19^. While our previous study did not find any significant effect of NFAT-inhibition on TNFα-induced expression of either target, investigators working in other endothelial cell systems have identified a regulatory role for NFAT in the expression of both^23^,^31^,^32^,^33^. Isoform-specific siRNA knockdown indicates that this inconsistency may have been due to counteracting effects of individual isoforms that were masked by inhibition of all four calcineurin-dependent isoforms with INCA-6. Both NFATc2 and NFATc4 knockdown significantly reduced TNFα-induced expression of *SELE*, while NFATc3 increased its expression. In the case of *ICAM1* expression, NFATc2 again inhibited TNFα-induction and NFATc1 knockdown increased the induction. Again, this example emphasizes the importance of isoform-specific manipulation of NFAT.

In order to test whether these isoform-specific findings have a functional impact on TNFα-induced leukocyte adhesion, we evaluated the effect of isoform-specific siRNA in a PPFC assay. In this assay, TNFα-stimulation increases the capacity of endothelial monolayers to capture and firmly adhere PBMC flowing over the monolayer under physiologic conditions. Previous studies using this technique have shown that siRNA and pharmacologic treatments that directly target leukocyte adhesion proteins such as VCAM1, E-Selectin, and ICAM1, reduce the ability of leukocytes to adhere to the monolayer^18^,^19^,^35^,^36^. Based on these findings, we hypothesized that transfecting monolayers with siRNA for specific NFAT isoforms shown to reduce TNFα-induced expression of these genes would lead to decreases in TNFα-induced PBMC adhesion. Accordingly, we found that transfection with NFATc2- and NFATc4-directed siRNA reduced TNFα-induced PBMC cell adhesion. One might expect that conversely, transfection with NFATc1 and NFATc3 siRNA, which, in some cases, increased TNFα-induced expression of these targets, might increase TNFα-induced PBMC adhesion, but neither NFATc1 nor NFATc3 knockdown had an effect on PBMC adhesion. The PPFC assay offers a number of interesting avenues for future studies based on these initial findings. For instance, combination treatments targeting the critical isoforms, NFATc2 and NFATc4, may provide an increased effect and should be considered moving forward. Additionally, while this study focused on the endothelial component of this assay, studies focused on the role of NFAT isoforms in the leukocyte component of this assay are particularly interesting. The whole PBMC used in this study includes both lymphocytes and monocytes, and it would be useful to determine which population of cells adhere to HRMEC in this assay, and what effect activation or a chronic disease state such as diabetes, has in this context.

Our previous analysis using INCA-6 also highlighted a role for NFAT-signaling in the regulation of TNFα-induced chemokines CXCL10 and CXCL11^23^. CXCL10 and CXCL11 both serve as ligands for the CXCR3 receptor and are known to play a role in leukocyte recruitment to sites of endothelial inflammation^24^,^25^,^26^. While leukocyte recruitment would not manifest functionally in our PPFC model of TNFα-induced leukocyte adhesion, leukocyte recruitment is a critical feature of TNFα-induced leukostasis *in vivo*. Accordingly, we measured the effect of isoform-specific siRNA on TNFα-induced protein secretion for each of these cytokines. In this experimental context, NFATc4 siRNA proved to be the only potent regulator of TNFα-induced cytokine secretion, inhibiting both CXCL10 and CXCL11. As was the case with E-selectin and ICAM1, MCP-1 and IL-6 are inflammatory products known to be upregulated by TNFα-stimulation and identified as NFAT-regulatory targets in other endothelial cell populations^32^,^34^. Thus, we included them in our evaluation and found that, while NFATc4 siRNA reduced MCP-1 levels in conditioned media, TNFα-induced secretion of IL-6 was unaffected by all isoforms. Taken together, these findings identify a strong role for NFATc4 in retinal endothelial cell production of leukocyte chemoattractants.

This role for NFATc4 highlights the importance of identifying isoform-specific effects and points toward an interesting line of future investigation regarding the mechanism that underlies these differential effects. The four isoforms evaluated in this study contain a similar, but not identical binding domain, and while it is tempting to attribute the unique expression profiles identified here to these differences, NFAT target gene expression is considerably more complex than direct binding. NFAT isoforms bind DNA as part of a dimer, and have been shown to be capable of acting as homo-dimers or hetero-dimers with other NFAT isoforms as well as additional transcription factors such as AP-1 and NFκB. This increased complexity means that slight variations in the DNA binding or dimerization domain between the individual isoforms can lead to a variety of protein interactions and likely accounts for the unique target gene profiles observed in the present study. Future studies identifying the binding partners and DNA-binding-sites for the critical NFAT isoforms downstream of TNFα will be necessary for understanding the complete mechanism of action.

As a final proof of concept we evaluated the effect of general NFAT inhibition via INCA-6 treatment, in a mouse model of TNFα-induced retinal leukostasis. Intraocular injection of TNFα leads to increased leukocyte adhesion to the vascular endothelium, and provides an acute model for a pathological feature of retinopathy that may take months or years to develop in diabetics. Using an intraocular dose of INCA-6 that had already proven efficacious in a murine model of retinal neovascularization, we found that non-isoform-specific CN/NFAT-inhibition significantly inhibited TNFα-induced leukocyte adhesion in this experimental context^37^. It is worth noting again that all of the *in vitro* studies presented here focused on the endothelial component of this pathology. NFAT was first characterized in T-lymphocytes and there is a rich body of literature studying the role of NFAT-signaling in leukocyte biology, which suggests that at least part of the *in vivo* effect may be due to NFAT-inhibition in other retinal cell types or circulating leukocytes^38^,^39^,^40^. This point emphasizes the potential of future studies focusing on the role of NFAT isoforms in relevant leukocyte populations, and the need to identify the unique roles of individual isoforms in this complex system.

Collectively, these studies show a clear role for NFAT-signaling in TNFα-induced retinal leukostasis. Given that vitreous levels of TNFα are increased in patients with DR, and that pathologic retinal leukostasis is a complicating factor of DR progression and pathology, NFAT may represent an attractive target for therapeutics aimed at TNFα-induced retinal leukostasis and DR. The isoform-specific effects identified in this study both highlight the need to target individual isoforms and identify NFATc2 and NFATc4 as potentially valuable therapeutic targets that warrant additional study.

## Materials and Methods

### HRMEC Culture

Primary cultures of HRMEC (Cell Systems; Kirkland, WA) were grown in tissue culture flasks coated with attachment factor (Cell Systems; Danvers, MA). Growth medium consisted of endothelial basal medium (EBM; Lonza; Walkersville, MD) supplemented with 10% FBS, 1× Antibiotic/Antimycotic solution (Sigma Aldrich; St. Louis, MO), and endothelial cell growth supplements (EGM SingleQuots; Lonza). All cultures were incubated at 37 °C, 5% CO_2_ and 95% relative humidity (20.9% oxygen), and passages 3 to 7 were used in these experiments.

### HRMEC Transfection

Control siRNA (Negative Control, Catalog no.: 1022076, Qiagen; Valencia, CA), NFATc1 siRNA (Mix of Hs_NFATC1_6 Catalog no.: SI03082422 and Hs_NFATC1_8 Catalog no.: SI03114902, Qiagen), NFATc2 siRNA (Hs_NFATC2_1 Catalog no.: SI00099512, Qiagen), NFATc3 siRNA (Hs_NFATC3_3 Catalog no.: SI00157997, Qiagen) and NFATc4 siRNA (NFATc4 siRNA (h), Catalog no.: sc-38115, Santa Cruz Biotechnology; Dallas, TX, USA) were used at 75 nM to transfect HRMEC as previously described^41^. Knockdown efficiency of siRNAs was confirmed by qRT-PCR analysis (Supplemental Fig. 1).

### Quantitative Real-Time RT-PCR (qRT-PCR) in HRMEC

HRMEC were seeded in 6-well plates at 2 × 10^5^ cells/well and maintained under growth conditions. At 75% confluence, HRMEC were transfected with control, NFATc1-, NFATc2-, NFATc3-, or NFATc4-directed siRNA as described above. Twelve hrs post-transfection, cells were transferred to 2% medium (medium containing 2% FBS) for 12 hrs, before being placed in 0.5% FBS medium for an additional 8 hrs. Cells were then stimulated with either 0.5% medium or 0.5% medium plus 1 ng/ml TNFα (Millipore; Billerica, MA) for 4 hrs. After treatment, cells were washed with cold phosphate-buffered saline (PBS; Invitrogen; Grand Island, NY) and total RNA was collected using an RNeasy kit (Qiagen), according to the manufacturer’s instructions. Total RNA was reverse transcribed using the High-Capacity cDNA Archive Kit (Applied Biosystems; Foster City, CA). qRT-PCR was performed by co-amplification of the gene of interest (*CX3CL1, VCAM1, SELE, or ICAM1*) vs *β-actin* (endogenous control) using gene-specific TaqMan Gene Expression Assays (*CX3CL1:* Hs00171086_m1; *VCAM1:* Hs01003372_m1; *SELE:* Hs00950401_m1; *ICAM1:* Hs00164932_m1; *β-actin:* Hs99999903_m1; Applied Biosystems). Expression data were analyzed using the comparative Ct method.

### Parallel Plate Flow Chamber Assay

HRMEC were grown to confluence on attachment factor-coated glass slides and transfected with control, NFATc1-, NFATc2-, NFATc3-, or NFATc4-directed siRNA as described above. Twelve hrs post-transfection, cells were switched to 2% medium for an additional 20 hrs, and 2% medium was used through out this experiment to maintain monolayer viability on glass slides. Cells were then stimulated with either fresh 2% medium or 2% medium plus 1 ng/ml TNFα for 4 hrs. After treatment, slides were mounted in a rectangular parallel plate flow chamber (GlycoTech; Gaithersburg, MD) with a flow width of 1.00 cm and chamber height of 0.005 in. Frozen PBMC (Sanguine Biosciences; Valencia, CA) were rapidly thawed and washed with endothelial cell growth medium and then resuspended in Hank’s Buffered Salt Solution (HBSS; Life Technologies; Carlsbad, CA) at a concentration of 5 × 10^5^ cells/ml. A syringe pump (World Precision Instruments; Sarasota, FL) was used to pull suspended PBMC across HRMEC monolayers at a shear stress of 1 dyn/cm^2^ for 7 min. Non-adherent cells were washed off with HBSS at 2 dyn/cm^2^ for 2 min. Eight fields of view were captured from each slide using an IMT-2 inverted microscope (Olympus; Tokyo, Japan) and Q-Color3 digital camera (Olympus) at 20× magnification, then manually counted by two masked observers. Adherent cell counts of all the captured fields of a single slide were averaged and reported as adherent cells per mm^2^. Experiments consisted of at least four independent HRMEC monolayers for each treatment group.

### Soluble Protein Quantification

HRMEC were seeded in 6-well plates and grown to 75% confluence, before being transfected with control, NFATc1-, NFATc2-, NFATc3-, or NFATc4-directed siRNA as described above. Twelve hrs post-transfection, cells were switched to 2% medium for 12 hrs, before being placed in 0.5% medium for an additional 6 hrs. Cells were then stimulated with either 0.5% medium or 0.5% medium plus 1 ng/ml TNFα for 6 hrs. After treatment, culture medium was collected and assayed for secreted CXCL10, CXCL11, MCP-1, and IL-6 protein concentrations using protein specific colorimetric sandwich ELISA kits (R&D Systems; Minneapolis, MN). Cells were washed with cold PBS and lysed using CellLytic lysis buffer (Sigma Aldrich), and the concentration of cell lysates was determined using a bicinchoninic acid assay (Pierce; Rockford, IL). Secreted protein concentrations were normalized to total protein of corresponding cell lysates and reported as pg/mg of total cellular protein.

### Retinal Leukostasis Assay

All experiments were approved by the Vanderbilt University Institutional Animal Care and Use Committee and were performed in accordance with the ARVO Statement for the Use of Animals in Ophthalmic and Vision Research. Six-week old male C57BL/6 mice were procured from Charles Rivers (Wilmington, MA). Mice received a 2 μl intravitreal injection of TNFα (50ng/ml) plus vehicle (0.1% DMSO in PBS) or INCA-6 (25 μM; Tocris; Minneapolis, MN). Six hrs later, mice were anesthetized with ketamine and xylazine and perfused at physiological pressure (between 100 and 122 mmHg for mice) with 0.9% saline for 6 min, followed by FITC-conjugated concanavalin-A (40 μg/ml in 2.5 ml PBS, Vector Laboratories; Burlingame, CA). Residual non-adherent leukocytes were washed out using an additional 6 min saline perfusion. Retinas were dissected in 4% paraformaldehyde, flat-mounted, and imaged with an AX70 upright scope (Olympus) and DP71 digital camera (Olympus) at 4× magnification, then lumenal leukocytes were manually counted by two masked observers. Retinal leukocyte counts for an entire retina were averaged and reported as retinal leukocytes per mm^2^. Each treatment arm consisted of at least 5 retinas.

### Statistical Analysis

Data were analyzed with JMP software (SAS Institute; Cary, NC) using One-Way ANOVA with Dunnett’s post hoc analysis. Values of p < 0.05 were considered statistically significant.

## Additional Information

**How to cite this article**: Bretz, C. A. *et al.* NFAT isoforms play distinct roles in TNFα-induced retinal leukostasis. *Sci. Rep.* **5**, 14963; doi: 10.1038/srep14963 (2015).

## Supplementary Material

Supplementary Information

## Figures and Tables

**Table 1:** Summary of the effects of isoform-specific siRNA on TNFα-induced induction in HRMEC.

	TNFα-Induction	NFATc1siRNA	NFATc2siRNA	NFATc3siRNA	NFATc4siRNA
Adhesion Protein Expression					
CX3CL1	266-fold	–	↓ 46%	↑ 90%	–
VCAM1	56-fold	–	↓ 35%	–	–
SELE	136-fold	–	↓ 44%	↑ 42%	↓ 37%
ICAM1	24-fold	↑ 29%	↓ 33%	–	–
PBMC Adhesion					
	11.5-fold	–	↓ 55%	–	↓ 38%
Secreted Cytokines					
CXCL10	4.1-fold	–	–	–	↓ 58%
CXCL11	2.8-fold	–	–	–	↓ 106%
MCP-1	2.4-fold	–	–	–	↓ 70%
IL-6	3.9-fold	–	–	–	–
