# Supplementary material for: NFAT isoforms play distinct roles in TNFα-induced retinal leukostasis
Source: Sci Rep. 2015 Nov 3;5:14963. doi: 10.1038/srep14963 (PMC4630625; doi:10.1038/srep14963)
Supplement: Supplementary Information [file srep14963-s1.pdf]

NFAT isoforms play distinct roles in TNFα-induced retinal leukostasis

Colin A. Bretz, PhD, Sara R. Savage, Megan E. Capozzi, Sandra Suarez, and John S. Penn, PhD

Supplemental Figure 1.

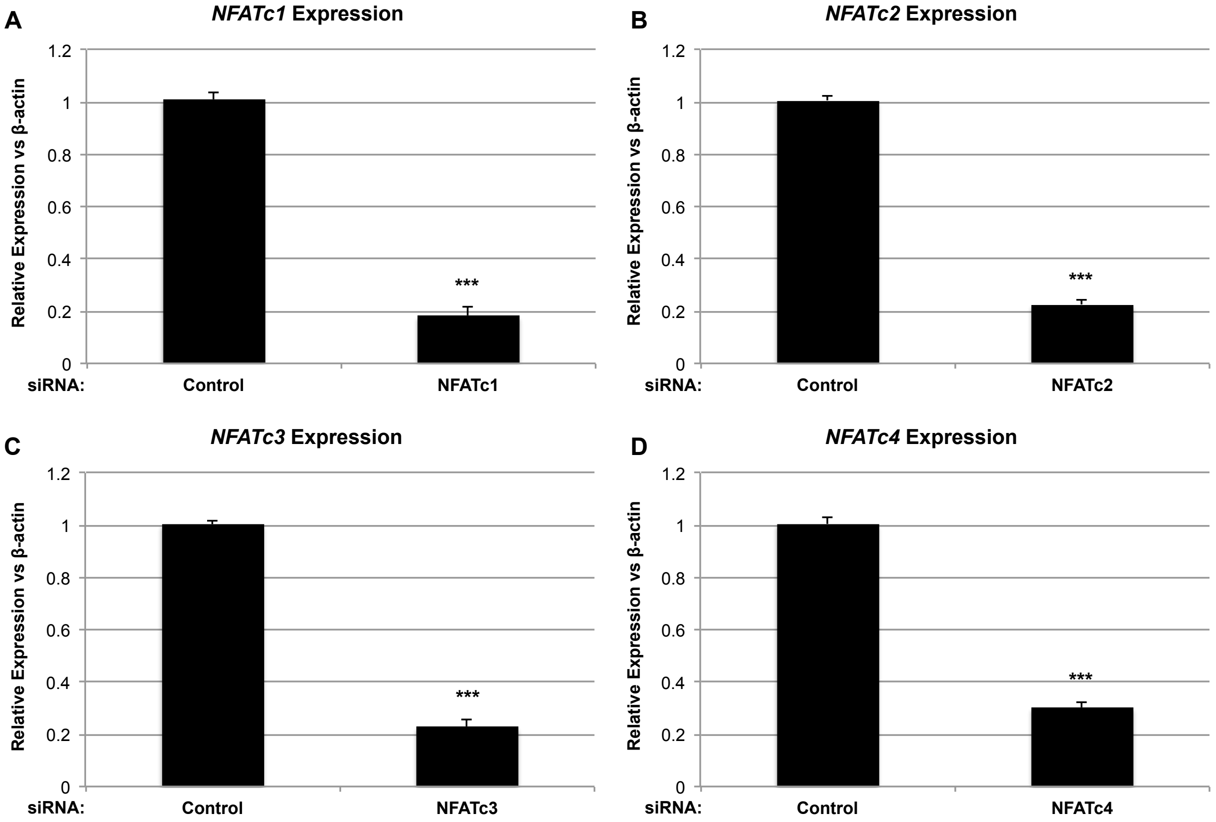

Supplemental Figure 1. The effect of NFAT isoform-specific siRNA on NFAT isoform expression in HRMEC. HRMEC were transfected with either control or NFAT isoform-specific siRNA, total RNA was collected and expression of NFAT isoforms was analyzed using qRT-PCR. **A) *NFATc1* Expression**. Transfection with NFATc1 siRNA inhibited *NFATc1* expression by 82.0%. **B) *NFATc2* Expression.** Transfection with NFATc2 siRNA inhibited *NFATc2* expression by 77.6%. **C) *NFATc3* Expression.** NFATc3 siRNA inhibited *NFATc3* expression by 77.3%. **D) *NFATc4* Expression.** NFATc4 siRNA inhibited *NFATc4* expression by 70.1%. Each bar represents the mean ± SEM (n=9). *** = $p<0.0001$
